# Effectiveness of Mechanical Horse-Riding Simulator-Based Interventions in Patients with Cerebral Palsy—A Systematic Review and Meta-Analysis

**DOI:** 10.3390/bioengineering9120790

**Published:** 2022-12-11

**Authors:** Esteban Obrero-Gaitán, Desirée Montoro-Cárdenas, Irene Cortés-Pérez, María Catalina Osuna-Pérez

**Affiliations:** 1 Department of Health Sciences, University of Jaen, Campus Las Lagunillas, 23071 Jaén, Spain; 2 Physiotherapy Service, FREMAP Mutua Colaboradora con la Seguridad Social nº 61, Santo Reino Street 7, 23003 Jaén, Spain; 3 Department of Nursing, Physiotherapy and Medicine, University of Almeria, Road Sacramento s/n, 04120 Almeria, Spain

**Keywords:** cerebral palsy, horse-riding simulator, gross motor function, balance, sitting, spasticity, range of motion, meta-analysis

## Abstract

Background: Mechanical horse-riding simulator (HRS) exercises are a type of therapy based on the use of robotic or mechanical devices that produces movement similar to a real horse with the aim of simulating hippotherapy. This review analyses the effectiveness of HRS therapies in patients with cerebral palsy (CP). Methods: A systematic review and a meta-analysis were carried out by searching studies in PubMed Medline, SCOPUS, Web of Science, CINAHL, PEDro and SciELO up until October 2022. We selected clinical trials that assessed the effectiveness of HRS therapy, compared to other interventions, in patients with CP. The main variables were gross motor function (its global score and dimensions, such as sitting ability), functional balance, spasticity, hip range of motion (ROM), posturographic balance and satisfaction. The risk of bias was assessed using the Cochrane Risk of Bias Tool. The pooled effect was calculated using Cohen’s Standardized Mean Difference (SMD) for a 95% confidence interval (95% CI). Results: Twelve studies were included in the systematic review, and 10 were included in the meta-analysis, providing data from 343 patients with spastic diplegic CP. Our findings revealed that HRS plus physiotherapy is more effective than physiotherapy in improving the total gross motor function (SMD 0.98; 95% CI 0.35–1.62), sitting ability of the gross motor function (SMD 0.84; 95% CI 0.32–1.36) and functional balance (SMD 0.6; 95% CI 0.1–1.08), and HRS therapy is better than sham to improve pelvic abduction ROM (SMD 0.79; 95% CI 0.21–1.37). Conclusions: Horse-riding simulator-based therapy is an effective therapy to improve gross motor function, functional balance and abduction pelvic ROM in children with CP, in comparison to physiotherapy or sham.

## 1. Introduction

Cerebral palsy (CP) is the most common physical disability in children, affecting 17 million people worldwide [1]. Currently, in high-income countries, the estimated prevalence is 1.6 cases per 1000 live births [2], which has decreased relative to previous prevalence data (two to three cases per 1000 in 2013) [3]. Cerebral palsy encompasses a group of permanent movement, posture and motor function disorders that changes with age as a result of damage to the developing fetal or infant brain [4,5]. Cerebral palsy is caused by brain damage that, among other effects, reduces activity in the motor cortex [6], producing inadequate design and execution of motor inputs and worse processing of corticospinal and somatosensory circuits [7]. Cerebral palsy is characterized by changes in musculoskeletal tissue, such as muscle weakness, muscle spasticity, decreased muscle strength, or restrictions in the shoulder and pelvic joint range of motion (ROM) [8,9,10,11]. These motor disorders limit the development of typical motor function and the acquisition of the necessary skills to ensure the maintenance of posture and balance, resulting in delayed onset of gait or the development of pathological gait patterns [12]. It is estimated that around 90% of children with CP exhibit gait difficulties [13]. In addition to alterations in standing-up posture during gait, the inadequate and unsafe sitting position is another inconvenience for these patients [14]. Balance, gait and sitting disorders related to motor impairments in children with CP reduce physical function and participation in activities of daily living [15], restricting interactions in social life fields such as leisure activities, education, self-care and social relationships [16]. Therefore, the recovery of assisted or non-assisted walking and gait efficiency are sometimes the main goals of physiotherapy interventions for these children in order to guarantee their functional independence [17].

Currently, there is a wide variety of medical, physiotherapeutic and animal-assisted therapy approaches to reduce motor, balance and gait disorders in children with CP [18]. The techniques that are commonly used to treat them are focused on early interventions that take advantage of the neuroplasticity of the brain [18]. From the point of view of medicine, the injection of botulinum toxin A stands out, which has been shown to be more effective in reducing spasticity and increasing ROM when it is applied in combination with physiotherapy [19]. Physiotherapy encompasses a wide variety of techniques to reduce the disability of these patients. These include neurodevelopmental therapies, such as Bobath [20]; conventional therapy based on mobilizations, stretching, functional therapeutic exercise and strength [21]; treadmill training and restraint-induced movement therapy [22]; or electrotherapy [23]. All of these therapies have been shown to be effective in improving gross motor function, balance, gait and functional capability in children with CP. In addition, technological advances have allowed new ways of performing physiotherapy techniques that increase patient motivation, thanks to virtual reality [24,25] or robotic devices [26], although the latter has not been shown to be more effective than physiotherapy.

As a complement to these therapies, hippotherapy represents a complementary novel approach used in children with CP. Hippotherapy or horse riding therapy is an equine-assisted therapy that uses horse movements in the rehabilitation of neurological diseases [27,28] due to the motor and sensory input it provides [29], which must be carried out under the guidance of a physiotherapist with hippotherapy qualifications [27]. Hippotherapy exercises focus on challenging the rider’s ability to maintain balance and sitting posture through the gait of the horse [30]. Some authors suggest that the repetitive and rhythmic movement of the horse imitates the 3-axial movement pattern of the trunk and pelvis during human gait [27,31]. Furthermore, this movement, combined with the warmth of the horse, is hypothesized to decrease spastic muscle tone in children with CP. Some studies show that hippotherapy improves motor and balance disorders in these children, although it raises doubts about whether its efficacy is superior to conventional physiotherapy [32]. Despite the reported benefits of hippotherapy, there are some drawbacks that justify why this therapy is not widely used in clinical practice. Some of them are the high costs of caring for horses, their training and the accessibility of patients to this therapy; the location and scarcity of hippotherapy centers; and its availability or the weather, among others [33]. To improve the patient’s accessibility to hippotherapy treatments, mechanical horse-riding simulators (HRS) have been developed in recent years so that the patient can receive rehabilitation without having to leave the physiotherapy consultation. An HRS is a type of intervention based on hippotherapy principles. HRSs are designed as a substitute for equine-assisted therapies in an attempt to make hippotherapy more accessible in a clinical setting [34]. An HRS mimics the passive movement of the horse-walking pattern through a robotic device with a dynamic saddle [35] and offers the advantage of enabling therapy with no spatiotemporal or weather-related constraints [36]. Although this device cannot completely replace the real hippotherapy experience, it provides stimuli very similar to the horse movement pattern. In addition, it has some unquestionable advantages, such as its safety, the possibility to exactly recreate the riding session in an indoor setting and its adaptability to the attributes of each patient [33].

To date, reviews have assessed the effect of HRS therapy in other neurological conditions such as stroke [35], musculoskeletal conditions such as back pain [37] and in older adults [38] with interesting findings. There is currently no systematic review or meta-analysis looking exclusively at the effect of HRS therapy compared to other therapies. In 2019, Dominguez-Romero et al. assessed the effectiveness of HRS therapy in patients with stroke and CP, including seven studies [35]. However, only four studies of all those included provided data from patients with CP and only two studies were used to perform the meta-analysis on gross motor function (total score) without finding statistically significant differences between HRS therapy and others. In this review, we found important restrictions, such as the use of the English language filter, the low number of studies included and that other variables such as balance or sitting ability were not assessed. In 2022, Heussen and Häusler, assessed the effectiveness of equine-assisted therapies for children with CP, including three studies that used HRSs [39]. This meta-analysis presents an important limitation; its literature search did not identify other studies that compare HRS therapy to other therapies in the outcome of interest. In addition, the generalization of these findings is difficult due to the low number of studies and the high risk of publication bias. To improve knowledge about the use of HRS in CP, the aim of this systematic review was to retrieve published evidence to assess the effectiveness of HRS interventions when comparing with other therapies in patients with CP on gross motor function, functional balance, spasticity, hip ROM, posturographical parameters and patients’ satisfaction. Secondarily, we determined if the effect of HRS therapy was bigger when it is used alone or combined with physiotherapy.

## 2. Materials and Methods

### 2.1. Register and Guidelines

This systematic review with meta-analysis was carried out following the recommendations of the Preferred Reporting Items for Systematic Reviews and Meta-Analyses (PRISMA) [40], the A Measurement Tool to Assess Systematic Reviews (AMSTAR version 2) [41], and the *Cochrane Handbook of Systematic Reviews of Interventions* (Second Edition) [42]. Furthermore, the protocol of this systematic review with meta-analysis was previously registered in the PROSPERO database, obtaining the following registration number: CRD42022370252.

### 2.2. Literature Search

Two authors (D.M.-C. and E.O.-G.) independently carried out a bibliographic search in the following databases: PubMed Medline, Web of Science (WOS), Scopus, CINAHL Complete, Physiotherapy Evidence Database (PEDro) and SciELO. In order to find studies not published in these databases, we searched in the reference section of studies previously published and in the gray literature (congress abstracts, proceedings, and documents of experts, among others). The search strategy was developed based on the PICOS tool proposed by the Cochrane Library [42,43]: population (children with CP), intervention (HRS), comparison (other therapies apart from HRS), outcomes (gross motor function, functional balance, spasticity, ROM and posturographic spine and balance parameters) and study design (clinical trials). Our search strategy was carried out using keywords from the PubMed Thesaurus (MeSH) and CINAHL Subject Headings. The main terms employed were “cerebral palsy” and “horse riding simulator,” and they were combined with other synonyms. The boolean operators were employed in our search strategy; “AND” was used to join the PICOS conditions selected, and “OR” to join related terms in each condition. Lastly, no language or publication date filters were used to perform the search. Any discrepancies related to the search were agreed with a third author experienced in literature searches (M.C.O.-P.). Table 1 shows the search strategy used in each database.

### 2.3. Inclusion and Exclusion Criteria: Study Selection

The selection process of the studies included in this meta-analysis was carried out by 2 authors (D.M.-C. and I.C.-P.) independently, who were responsible for reviewing all the records found in each database by title and abstract. In addition, discrepancies in this phase were resolved by a third author (M.C.O.-P.). A study was only included in this review if it met all the inclusion criteria: (1) clinical trials; (2) that the study population was diagnosed with CP; (3) that the study had at least 2 intervention groups, 1 of which underwent HRS therapy and it was compared to another type of therapy different to HRS or no intervention; (4) studies that evaluated variables of interest for this study (see Section 2.5); and (5) studies that provided qualitative or quantitative data to perform the qualitative synthesis or meta-analysis. The following exclusion criteria were also established: (1) clinical trials where the sample comprised patients with different neurological diseases (not only CP); and (2) experimental studies with only 1 group (without a comparison group).

### 2.4. Data Extraction

The data extraction process of the included studies was carried out by two authors (D.M.-C. and E.O.-G.) independently, using a Microsoft Excel data collection form. All possible disagreements were resolved with a third author (M.C.O-P.). The following data were extracted from each study: (1) general characteristics (authorship, publication date, study design, country, setting and funding); (2) patient characteristics (total sample size, number of participants per group, age, sex, type of CP, disability and time since diagnosis); (3) characteristics of the experimental group and the control group (type of intervention, number of sessions, number of weeks, number of sessions per week, and duration of each session in minutes); (4) outcome data of the variables of interest (mean and standard deviation if a meta-analysis and qualitative synthesis and p-value for intra-groups and inter-groups comparisons); and (5) assessment time (post-intervention). When a study did not provide standard deviations, it was estimated using standardized transformations through the standard error, range, interquartile range and median [42,44].

### 2.5. Variables

The variables of interest to assess the effectiveness of HRS therapy in patients with CP were gross motor function and its five dimensions (lying and rolling, sitting, crawling and kneeling, standing up and gait ability), functional balance, spasticity, hip ROM and posturographic parameters and satisfaction.

### 2.6. Quality Assessment

The evaluation of the risk of bias in each study included and of the quality of evidence of the main findings was carried out by 2 authors (D.M.-C. and M.C.O.-P.) independently. Any doubts were resolved by a third author (E.O.-G.). At first, the Cochrane Collaboration Bias Tool Risk was used to assess the risk of bias in the studies included in the review. This scale assesses 6 bias domains (selection, performance, detection, attrition, reporting and others) through seven items (random sequence generation, concealment randomization sequence, blinding of participants, blinding of assessors, incomplete outcome data, selective reporting and others, ideally prespecified). Each item can be categorized as “+” (high risk of bias), “−“ (low risk of bias), and “?” (uncertain risk of bias) [45]. Secondly, the quality of evidence of each meta-analysis was assessed using the *Grading of Recommendations Assessment, Development and Evaluation* (GRADE) [46]. Furthermore, the recommendations of the checklist proposed by Meader [47] were followed to estimate the quality of the evidence, taking into account the risk of bias in each selected study, the inconsistency, the imprecision, the lack of directivity and the risk of publication bias. The quality of evidence was categorized as high (if our findings were robust); moderate (if our results changed when introducing new studies); low (if our results were very slight); and very low (when some elements were not present). The quality of the evidence for each meta-analysis was downgraded by one level for each factor found. When multiple limitations were found, the overall quality score was lowered by 2 levels.

### 2.7. Statistical Analysis

The meta-analysis was carried out by 2 authors (E.O.-G. and I.C.-P.) using the software Comprehensive Meta-Analysis v3.0 (Biostat, Englewood, NJ, USA) [48]. To perform the meta-analysis, we followed the recommendations of the *Introduction to Meta-Analysis* by Borenstein et al. [49] and of *The Handbook of Research Synthesis and Meta-Analysis*” by Cooper et al. [50]. According to the level of heterogeneity in each meta-analysis, we used a random or fixed effect model in accordance with Dersimonian and Laird [51]. Cohen’s standardized mean difference (SMD) and its 95% confidence interval (95% CI) were used to calculate the pooled effect [52]. Effect size could be null (SMD 0), low (SMD 0.1–0.39), moderate (SMD 0.4–0.79) or large (SMD > 0.8) [53]. Additionally, when the same variable was measured with the same tests, we calculated the mean difference (MD) and its 95% CI, with the aim of comparing this result to the minimally clinically important difference (MCID) value for this test. Jaeschke et al. defined the MCID as “the smallest difference in score in the domain of interest which participants perceive as beneficial and which would mandate, in the absence of troublesome side effects and costs, a change in the patient’s management” [54]. The findings of each meta-analysis were graphically represented in the forest plots [55]. The risk of publication bias was assessed taking into account three elements: the visualization of the funnel plots (asymmetry indicates the presence of risk of publication bias) [56]; the *p*-value for the Egger test (*p* < 0.1 indicates the risk of publication bias) [57]; and the trim-and-fill estimation [58,59]. If variations were found after trim-and-fill estimation that was larger than 10% of the original pooled effect, the quality of evidence would be downgraded by 1 level, even though the funnel plot was symmetric [60]. Finally, the level of heterogeneity was calculated using the degree of inconsistency (I^2^) and the *p*-value for the Q-test (*p* < 0.01 indicates the risk of heterogeneity). The heterogeneity could be null (I^2^ 0%), low (I^2^ < 25%), moderate (I^2^ 25–50%) or large (I^2^ < 50%) [61,62].

### 2.8. Additional Analyses

To assess the contribution of each study to the overall pooled effect, we performed a sensitivity analysis using the leave-one-out method. In addition, we performed the following subgroup analyses: HRS plus PT (physiotherapy) vs. PT, HRS vs. PT, and HRS vs. sham.

## 3. Results

### 3.1. Study Selection

**Figure 1:**
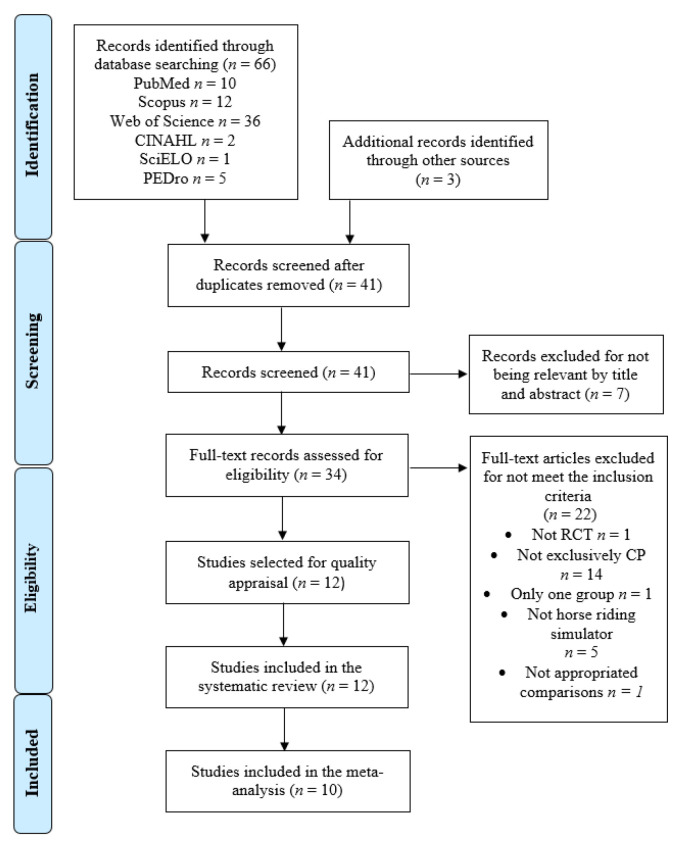
PRISMA flow diagram.

The PRISMA flow chart (Figure 1) shows the study selection process. Initially, 69 records were retrieved from the initial bibliographic search (66 from the databases and three from other sources). After removing 28 duplicates and seven records as not relevant by title and abstract (HRS or CP were not the major topics of these studies), 22 articles were assessed for eligibility by applying the inclusion criteria. Twenty-one studies were deleted for not meeting the inclusion criteria (reasons in Figure 1). Finally, 12 clinical trials were included in this review [63,64,65,66,67,68,69,70,71,72,73,74]. All studies provided data for the qualitative synthesis (systematic review), and 10 reported quantitative data for use in the quantitative synthesis (meta-analysis) [63,64,67,68,69,70,71,72,73,74].

### 3.2. Characteristics of the Studies Included in the Review

The studies included in this review were conducted between 1998 and 2022 in India [64,67], South Korea [65,66,70,71], Iraq [63], Spain [69], Brazil [73], Thailand [74] and the USA [72]. The included studies reported data from 343 patients with spastic diplegic CP with ages between 2 and 16 years old, of which 56% were male versus 44% female. The experimental group comprised 180 patients who received an HRS intervention alone [68,69,72,73,74] or in combination with conventional therapy [63,64,65,66,67,70,71]. The control group comprised 163 patients who received conventional therapy [63,64,65,66,67,70,71,73] or sham [68,69,72,74]. The duration of HRS therapy ranged from 1 to 12 weeks. The sessions were carried out one to three times per week, with the duration of each session ranging from 10 to 75 min. There was no follow-up in any of the studies, and all assessments were performed at the end of the intervention. Finally, only one study received external funding [69]. Table 2 shows the characteristics of the included studies.

### 3.3. Risk of Bias Assessment

Table 3 shows the Cochrane Risk of Bias Tool assessment for each study included in the review. The risk of bias was high in three studies [65,66,70], medium in six studies [63,64,67,71,73,74], and low in three studies [68,69,72]. The most important risks for consideration were performance, detection and selection biases. The risk of performance bias was present in all studies due to the impossibility of blinding the participants. Detection bias appeared in seven studies (58% of all), and selection bias was present in six studies (50% of all studies).

### 3.4. Variables, Measurements and Synthesis

To assess gross motor function, the studies included reported data from the Gross Motor Function Measure-66 (GMFM-66), GMFM-88 and Gross Motor Function Classification System (GMFCS). To analyze gross motor function, we obtained data from the total score and/or its five dimensions (A: lying and rolling; B: sitting; C. crawling and kneeling; D: standing; and E: walking, running and jumping). Secondly, postural balance was assessed with data from the Pediatric Balance Scale (PBS). Thirdly, spasticity in different lower limb muscles was assessed with data from the Modified Modified Ashworth Scale (MMAS) and Modified Ashworth Scale (MAS). Later, hip and tilt pelvic ROM was evaluated with a goniometer.

Furthermore, other secondary variables included posturographic parameters of static balance using Pedoscan Sensor and F-mat sensor platform and F-scan system, posturographic spinal posture using ABW Mapper, seated trunk control with SATco and satisfaction with the therapy using the Autoquestionnaire Qualité de Vie Enfant image (AUQEI).

The results of this review are presented in two ways: first, a meta-analysis of the variables gross motor function, functional balance, spasticity and ROM and, then, a qualitative synthesis for pelvic tilt, posturographic balance assessment in stand-up or sitting positions (trunk control) and satisfaction with HRS therapy. Table 4 shows all the qualitative findings in the studies included.

### 3.5. Quantitative Synthesis

Table 5 shows the main findings of the meta-analyses.

#### 3.5.1. Gross Motor Function

For gross motor function, we assessed the effect of HRS therapy on each dimension (A, B, C, D and E) and on the global total score.

**Figure 2:**
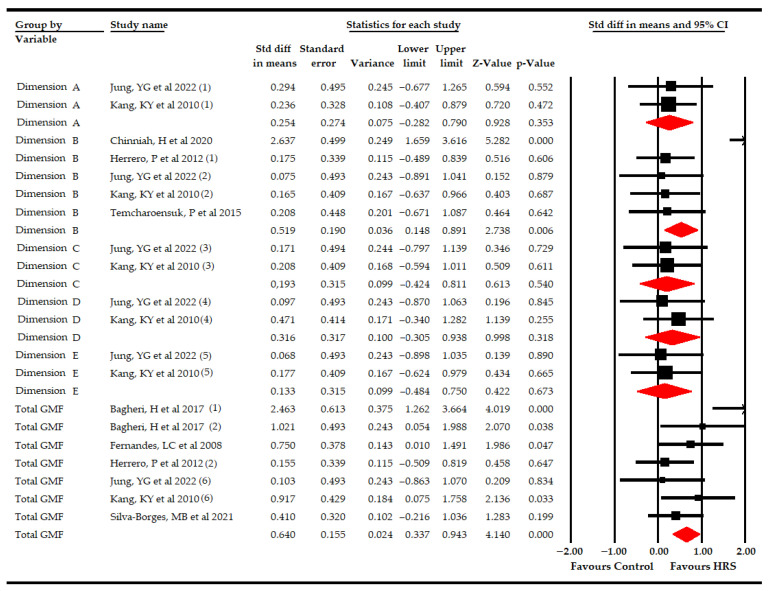
Forest plot of the effect of a Horse-Riding Simulator (HRS) therapy on gross motor function.

At first, two studies [70,71] reported data from 41 participants (20.5 per comparison) to assess the effect of HRS therapy on A, C, D and E dimensions of gross motor function. Our findings did not show statistically significant differences between HRS plus physiotherapy and physiotherapy on the A (SMD 0.25; 95% CI −0.28–0.79; *p* 0.35), C (SMD 0.19; 95% CI −0.42–0.81; *p* 0.54), D (SMD 0.32; 95% CI −0.31–0.94; *p* 0.32) and E dimensions (SMD 0.13; 95% CI −0.48–0.75; *p* 0.67; Table 5, Figure 2). The risk of publication bias could not be studied, and no heterogeneity was present.

**Figure 3:**
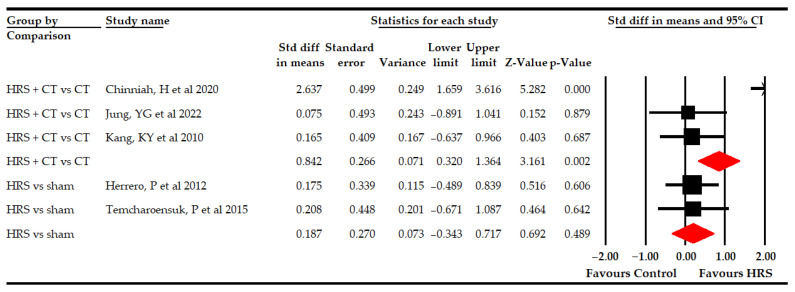
Subgroup analyses of the effect of Horse-Riding Simulator (HRS) therapy on sitting ability.

Secondly, five studies [64,69,70,71,74] reported data from 129 patients (25.8 per comparison) to assess the effect of HRS therapy on the B dimension (sitting ability) of gross motor function. Our findings showed a medium effect (SMD 0.52; 95% CI 0.15–0.9; *p* 0.006) favors HRS (Table 5, Figure 2). A low risk of publication bias was present (*p* for Egger 0.07) due to trim-and-fill and showed a variation of 15% (adjusted SMD 0.6; 95% CI 0.24–0.94) with respect to the original pooled effect (Figure S1). The level of heterogeneity was moderate (I^2^ 41%; *p* 0.04). Subgroup analysis revealed that the use of HRS plus physiotherapy was better (SMD 0.84; 95% CI 0.32–1.36; *p* 0.002) than physiotherapy alone, showing an improvement in the sitting ability of the GMF-88 test of 7.64 points (95% CI 0.41–14.82; *p* 0.038). However, no statistically significant differences were found between HRS vs. sham (SMD 0.19, 95% CI −0.34–1.36; *p* 0.49; Table 5, Figure 3).

**Figure 4:**
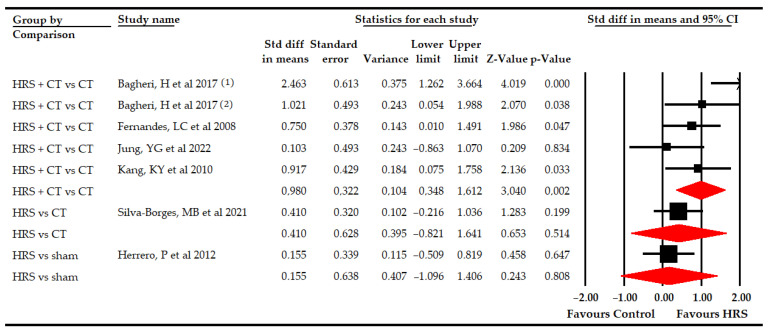
Subgroup analyses of the effect of Horse-Riding Simulator (HRS) therapy on the total score of the gross motor function.

Finally, six studies [63,67,69,70,71,74] with seven independent comparisons provided data from 187 participants (26.7 per comparison) to assess the effect of HRS therapy on the total score of gross motor function. Our findings reported a medium effect (SMD 0.64; 95% CI 0.34–0.94; *p* < 0.001) in favor of HRS therapy (Table 5, Figure 2). The risk of publication bias was not present, and heterogeneity was low (I^2^ 10.7%; *p* 0.35). A subgroup analysis revealed a large effect (SMD 0.98; 95% CI 0.35–1.62; *p* 0.002) favoring HRS plus physiotherapy vs. physiotherapy (Figure 4 and Figure S2 for its risk of publication bias), improving the GMF-66 by 7.36 points (95% CI 2.91–11.8; *p* 0.001) and the GMF-88 by 11.21 points (95% CI 0.85–21.57; *p* 0.034). However, no statistically significant differences were found between HRS vs. physiotherapy (SMD 0.41; 95% CI −0.82–1.64; *p* 0.52) and HRS vs. sham (SMD 0.15; 95% CI −1.01–1.4; *p* 0.8; Table 5, Figure 4).

#### 3.5.2. Functional Balance

**Figure 5:**
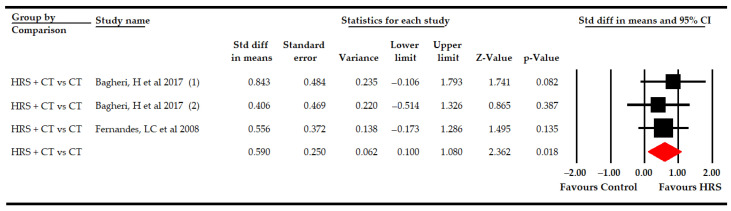
Forest plot of the effect of Horse-Riding Simulator (HRS) therapy on functional balance.

Two studies [63,67] with three independent comparisons provided data from 68 participants (22.7 per comparison) to assess the effectiveness of HRS therapy on functional balance. Our findings showed low-quality evidence of a medium effect (SMD 0.6; 95% CI 0.1–1.08; *p* 0.018) of HRS plus physiotherapy in comparison to physiotherapy (Table 5, Figure 5). In addition, the combination of HRS plus physiotherapy increased the functional balance measured with PBS by 6.21 points (95% CI 1.14–10.62; *p* 0.015). No risk of publication bias or heterogeneity was found. Sensitivity analysis did not report substantial variations in the pooled effect when the studies were excluded.

#### 3.5.3. Abduction Pelvic Range of Motion

**Figure 6:**
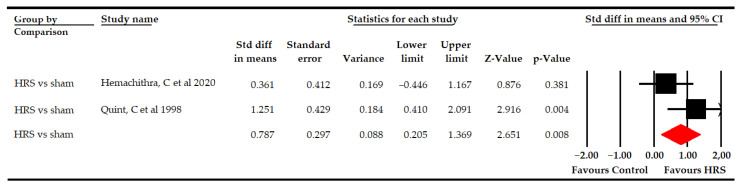
Forest plot of the effect of Horse-Riding Simulator (HRS) therapy on abduction pelvic range of motion.

Two studies [68,72] with two independent comparisons provided data from 54 participants (27 per comparison) to compare the effectiveness of HRS vs. sham in increasing abduction pelvic ROM. Our findings revealed a large effect (SMD 0.79; 95% CI 0.21–1.37; *p* 0.008) that favored HRS therapy (Table 5, Figure 6), being able to increase it by 7.49 degrees (95% CI 2.45–12.5; *p* 0.004), compared to sham. The risk of publication bias could not be studied, and heterogeneity was moderate (I^2^ 37%; *p* 0.13).

#### 3.5.4. Spasticity

The effect of HRS therapy in reducing spasticity was assessed in hip adductors, knee extensors and ankle dorsiflexors.

**Figure 7:**
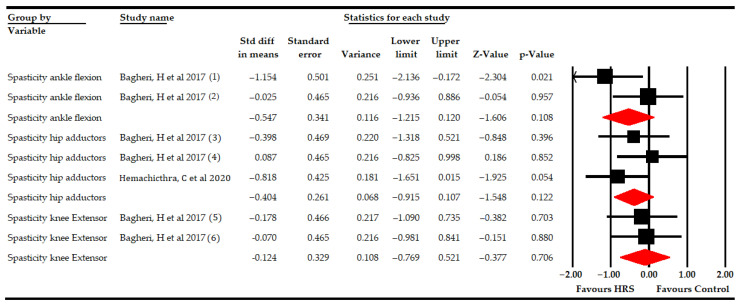
Forest plot of the effect of Horse-Riding Simulator (HRS) therapy on lower limb muscle spasticity.

**Figure 8:**
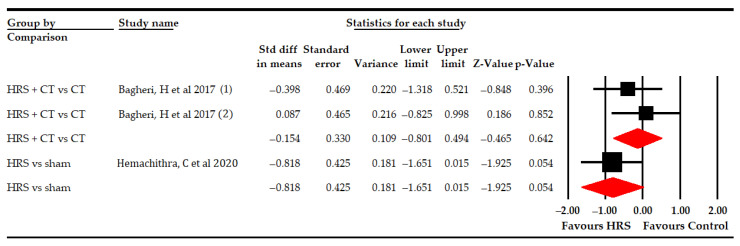
Forest subgroup analyses of the effect of Horse-Riding Simulator (HRS) therapy on hip adductors spasticity.

At first, two studies [63,68] with three independent comparisons provided data from 62 participants (20.7 per comparison) to assess the effect of HRS therapy on hip adductors spasticity without finding statistically significant differences between HRS therapy and the controls (SMD −0.4; 95% CI −0.92–0.11; *p* 0.122; Table 5, Figure 7). However, the risk of publication bias found was very large after the trim-and-fill estimation, estimating that without risk of publication bias, the statistically significant differences found would favor HRS therapy (adjusted SMD −0.81; 95% CI −1.21–−0.41; Figure S3). Heterogeneity was not present. Subgroup analysis revealed no statistically significant differences between HRS plus PT vs. PT (SMD −0.15; 95% CI −0.8–0.5; *p* 0.642) and between HRS vs. sham (SMD −0.82; 95% CI −1.65–0.02; *p* 0.054; Table 5, Figure 8).

Finally, the spasticity of the ankle dorsiflexors and of the knee extensors was assessed in one study [63] with two independent comparisons (each one) (Table 5, Figure 7). No statistically significant differences were found between HRS plus PT vs. PT in the reduction of the spasticity of the ankle dorsiflexors (SMD −0.55; 95% CI −1.22–0.12; *p* 0.11) or of the knee extensors (SMD −0.12; 95% CI −0.77–0.52; *p* 0.71).

### 3.6. Qualitative Synthesis

Regarding the qualitative synthesis of the studies included in our systematic review, we were able to reach the following conclusions. Two studies [65,72] compared the effect of HRS therapy on pelvic tilt. Despite using different treatment protocols, the results of both studies concluded that there were statistically significant improvements in those patients who received HRS therapy (*p* < 0.05). Furthermore, we found two studies [65,74] that compared the effect of HRS therapy on trunk control during sitting. Both studies concluded that there were greater statistically significant improvements in children who were part of the HRS therapy group, especially in improving reactive trunk control (*p* 0.004). Choi H.J. et al. (2014) and Silva-Borges et al. (2011) found statistically significant differences in medial-lateral sway [66,73] in the HRS therapy groups, while only Silva-Borges et al. (2011) found statistically significant differences between groups in anteroposterior sway [73]. Lastly, a single study [73] analyzed the satisfaction perceived by patients after treatment sessions. The study concluded that children who received treatment with HRS therapy perceived greater satisfaction compared to children who did not receive this therapy. In addition, this study reported that no child was unhappy with the use of the HRS, while 25% of children belonging to the CT group were unhappy with the therapy. Findings of the qualitative synthesis in each study included are shown in Table 4.

## 4. Discussion

Although the use of HRSs in the management of motor, balance and gait disorders in different neurological and musculoskeletal diseases is increasing in physiotherapy approaches, only six reviews have compiled the published evidence about HRS therapy on chronic pain [33,75], autism spectrum disorder [76], stroke and cerebral palsy [35,39] or older adults [38]. Due to the high prevalence of CP in children and the fact that CP produces a high level of disability in them, it is necessary to assess the effectiveness of the therapies applied to them, such as HRS or physiotherapy, and to analyze what therapy can provide more improvement in its recovery. There is no review that compiles all published articles that have assessed the effectiveness of HRS therapy in improving gross motor function, balance, spasticity or hip ROM in children with CP. Two previous reviews [35,39] have reported on the effect of HRS therapy on gross motor function or balance in these patients, but the number of studies included was less than four in each case, so these results may not be generalizable and are susceptible to change when new studies are included in the meta-analysis, due to a possible risk of publication bias. Therefore, our systematic review is the first meta-analysis that includes the largest number of studies to date to analyze the effect of HRS therapy in children with CP. In addition, our review assesses, for the first time, variables such as spasticity, hip ROM, posturographic parameters and the level of satisfaction of the children with CP with HRS therapy. In addition, when it was possible, we provided subgroup analyses to assess if the effect of HRS therapy is large when it is used alone or combined with physiotherapy. The findings of our meta-analyses show that HRS therapy is effective in improving gross motor function, functional balance and hip abduction ROM in children with CP.

Regarding gross motor function, we assessed the effect of HRS therapy on the total score and on each dimension. We found statistically significant differences favoring HRS therapy in improving sitting ability and total gross motor function in comparison to other therapies such as physiotherapy or sham. In addition, the effectiveness of HRS therapy on sitting ability (SMD 0.84) and total gross motor function (SMD 0.98) was large when it was used in combination with physiotherapy compared to physiotherapy. Our meta-analysis identified two important benefits of HRS plus physiotherapy on these variables. On the one hand, the effect of HRS plus physiotherapy was unknown until this study was conducted. On the other hand, we calculated the mean difference between therapies, showing that HRS plus physiotherapy was able to increase the sitting ability and global total score in the GMF-88 test by 7.64 points and 11.21 points, respectively, compared with physiotherapy. Currently, no study has published the MCID for sitting ability in this test, so our data cannot be compared to this MCID, but we consider that an increase of almost 8 points for sitting ability may have relevance for clinical practice, helping these patients to better carry out their activities of daily living in the sitting position. However, on the global total score, our findings exceed the MCID value in GMF-88, calculated in 2020 by Storm et al. [77]. These two findings represent the most important findings of our review and establish that HRS therapy is effective for improving sitting ability and gross motor function in children with CP, but the effect was more pronounced when HRS was used with physiotherapy. These findings cannot be compared with the review of Heussen and Häusler, as it does not report specific data for the effect of HRS therapy unless it is integrated into an analysis of hippotherapy and therapeutic riding [39].

Secondly, our findings showed that the inclusion of HRSs in physiotherapy protocols is effective for improving functional balance in comparison to physiotherapy only (SMD 0.6). We determined that the combination of HRS plus physiotherapy increased the total score in PBS by 6.21 points. Chen et al. (2013) reported that the MCID value for the total score of PBS in children with CP was 5.83 points [78]. Our findings showed that the use of HRSs in physiotherapy for recovering functional balance is clinically relevant, and it exceeds the MCID for PBS in children with CP [78]. In addition, this data is supported by improvements obtained in balanced sitting posturographic parameters; therefore, HRS therapy increases the sitting trunk control [65] and reduces medial-lateral [66] and anteroposterior sway area [73] when assessed with static posturography. Our results cannot be directly compared with previous reviews since there are no studies that analyze the effect of HRS therapy on functional balance. Only, Dominguez-Romero et al. (2020) found that HRS therapy was better than conventional therapy interventions in improving functional balance (Berg Balance Scale) in patients with stroke, using data from two studies for the meta-analysis, agreeing with them that HRS therapy is effective for improving functional balance in patients with central nervous system diseases [35]. Previous studies have shown that balance training on unstable support surfaces produces activation of the trunk musculature and a continuous response of the back muscles to maintain a stable center of mass [79,80]. The new HRS devices can generate specifically three-dimensional slight movements of the trunk and pelvis per minute, similar to those experienced by the body riding a real horse in hippotherapy and favoring the training of postural reactions of the trunk [10].

The last meta-analysis assessed the effectiveness of HRS therapy on abduction hip ROM and lower limb spasticity. Our results showed that HRS therapy was effective in increasing the hip abduction ROM by 7.5 degrees in a goniometry assessment, in comparison to sham. Regarding spasticity, HRS plus physiotherapy was not better than physiotherapy only in reducing hip adductor, knee extensor and ankle dorsiflexor spasticity. However, in the hip adductor spasticity meta-analysis, our findings were underestimated as a result of publication bias, and when the pooled effect was calculated, taking into account this possible bias (trim-and-fill variation of 100%), we found that HRS therapy could have a large effect (adjusted SMD −0.81) in reducing spasticity in adductor muscles. This last result highlights the importance of performing future research with the aim of confirming this possible finding without the risk of publication bias. The improvement in hip abduction ROM and possible reduction in adductor spasticity can be explained by the continuous riding position of the equine simulator, in which both hips are abducted, keeping the adductor muscles continuously stretched, reducing its shortening and helping to prevent the neuromuscular hip dysplasia which is common in these children [81].

Finally, our review highlights an important finding in the study of Silva–Borges et al. (2011), in which the level of satisfaction in the HRS therapy was assessed in comparison to conventional therapy [73]. Children with CP that received HRS therapy reported more happiness, while in the conventional therapy group, approximately 25% of those children were unhappy with the therapy. This data highlights the need for therapies that attract children’s attention, thus increasing their motivation and adherence to therapy to obtain better results. In previous studies, patients with various pathologies who have been subjected to conventional classical treatments have shown signs of monotony and lack of adherence [82,83], which could explain why they did not experience a clear improvement compared to other more active and striking therapies, such as HRS, virtual reality-based therapy or robot-assisted gait training devices.

HRSs have some benefits that allow their easy inclusion within the clinical practice, including the lower costs of maintenance of the machine compared to the costs of care and training of horses [33,75]. Moreover, the facilities where HRS therapy is carried out do not have to be large, unlike the facility that is needed to carry out therapy with real horses. Due to the large size requirements, many of the centers where hippotherapy is carried out are outside of urban centers, which means an extra cost for the trip to the facility for families. An HRS, being a device of relatively small size, can very easily be part of a hospital or neurorehabilitation clinic. There are also other aspects that favor therapy with HRSs, such as weather conditions, children’s fear of riding the animal or potential allergic reactions that children may develop [75], although it also can appear due to plastic or metal materials of HRS being built.

Although the findings presented in this systematic review and meta-analysis are interesting and relevant for clinical practice, it is important to note some limitations. First, the small number of studies that are included for each variable in which the meta-analysis has been carried out should be highlighted. This is not a limitation of our literature search process unless, due to the scarcity of studies that assess the effect of HRS therapy in CP that have been published to date, they did not meet the inclusion criteria. The second is related to the precision of the results, which is derived from the number of participants. The included studies comprise small sample sizes, and this may lead to underestimation of the results when combining the studies with meta-analysis, as has occurred. The low number of studies conducted to date and their small sample size may make it difficult to generalize the results and reduces the quality of the evidence from the overall analyses; however, no other reviews have been published to date. Third, it is important to highlight the moderate risk of bias in the included studies. Furthermore, the heterogeneity in the HRS therapy protocols used (frequency, number of sessions, and devices used) does not help determine which type of HRS therapy is the most effective. It is also important to highlight the risk of publication bias present in some studies. However, at this point, it is necessary to point out that the risk of publication bias meant that there were no statistically significant differences between HRS plus physiotherapy vs. physiotherapy in increasing spasticity hip adductors, and the trim-and-fill estimation determined that, without publication bias, HRS therapy would improve this variable. Finally, the last limitation is that this review only assessed the immediate effect of HRS therapy because the studies included did not provide follow-up data.

## 5. Conclusions

This is the first systematic review with a meta-analysis that quantitatively assesses the effect of HRS therapy on gross motor function, functional balance, spasticity and hip ROM and reports a qualitative synthesis of other secondary outcomes such as posturographic parameters and satisfaction of the participants, in comparison to physiotherapy or sham. Our meta-analysis reported that HRS therapy is effective in improving overall gross motor function and sitting ability, functional balance and hip abduction ROM in children with CP. More specifically, we reported that when HRS is combined with physiotherapy, the improvements found in gross motor function, sitting ability and functional balance are higher, but no differences between these therapies were found to reduce spasticity in hip adductors, knee extensors or ankle dorsiflexors. More studies that assess the effectiveness of HRS therapy in these variables and in others, such as quality of life or functional independence, are needed to carry out in the future. An increase in the sample size of these studies will help obtain more robust and accurate results and enable the HRS therapy findings to be generalized. Finally, to guarantee the comparison between therapies in future studies, it would be necessary to homogenize the duration and intensity of the HRS protocols and to evaluate the variables in different follow-up times, in addition to immediate post-intervention, to verify the efficacy of HRS over time.

## Figures and Tables

**Table 1:** Literature search strategies.

Databases	Search Strategies
PubMed Medline	(Horse riding simulator[tiab] OR horse-riding simulator[tiab] OR horse simulator[tiab] OR simulator horse[tiab] OR horse virtual[tiab] OR simulator equine[tiab] OR virtual reality horse simulator[tiab]) AND (cerebral palsy[mh] OR cerebral palsy[tiab] OR infantile cerebral palsy[tiab])
SCOPUS	(TITLE-ABS-KEY (“cerebral palsy”) AND TITLE-ABS-KEY (“Horse riding simulator” OR “horse simulator” OR “simulator horse” OR “simulator equine”))
Web of Science	TOPIC: (*cerebral palsy*) AND TOPIC: (*Horse riding simulator* OR *horse simulator* OR *simulator horse* OR *simulator equine*)
CINAHL Complete	AB (cerebral palsy) AND AB (Horse riding simulator OR horse simulator OR simulator horse OR simulator equine)
PEDro	Cerebral palsy AND horse riding simulator
SciELO	Cerebral palsy AND horse riding simulator

**Table 2:** Characteristics of the studies included in the review.

Study	Pathology			Experimental Intervention				Control Intervention			
	CP Type	GMFCS	N	N_e_	Age	F:M	Intervention	N_c_	Age	F:M	Intervention
Bagheri, H et al., 2017 (Iraq) [63] Setting: Medical Rehabilitation and Rheumatology Center, Baghdad, Iraq Funding: No	Spastic diplegic CP	II–III	30	11	4–13 years old	4:7	HRS plus strengthening training 8 weeks, 3 sessions per week, 75 min per session (15 min HRS plus 60 min of abdomen, back and lower limb strengthening exercises)	8	4–13 years old	3:5	Conventional therapy. 8 weeks, 3 sessions per week, 60 min per session.
				11	4–13 years old	5:6	HRS plus conventional therapy 8 weeks, 3 sessions per week, 75 min per session (15 min HRS plus 60 min of conventional therapy)				
Chinniah, H et al., 2020 (India) [64] Setting: Deparment of Physical Medicine and Rehabilitation, Annamalai Nagar, Tamil Nadul Funding: No	Spastic diplegic CP	I–III	30	15	2–3 years old	10:5	HRS plus conventional therapy 12 weeks, 3 days per week for 45 min per session (15 min of HRS plus 30 min of conventional therapy)	15	2–3 years old	7:8	Conventional therapy 12 weeks, 3 days per week, for 30 min per session.
Choi, HJ et al., 2014a (South Korea) [65] Setting: Suncheon Pyungwha Hospital Funding: No	Spastic diplegic CP	I–IV	30	15	8.8 ± 3.1 years old	4:11	Neurodevelopmental treatment plus HRS 10 weeks, 4 sessions per week, 45 min per session (30 min Neurodevelopmental treatment plus 15 min HRS)	15	9.3 ± 3.8	5:10	Neurodevelopmental treatment 10 weeks, 4 sessions per week, 30 min per session
Choi, HJ and Nam, KW 2014b (South Korea) [66] Setting: Suncheon Pyungwha Hospital Funding: No	Spastic diplegic CP	I–IV	30	15	8.8 ± 3.14 years old	4:11	Neurodevelopmental treatment plus HRS 10 weeks, 4 sessions per week, 45 min per session (30 min Neurodevelopmental treatment plus 15 min HRS)	15	9.27 ± 3.8 years old	5:10	Neurodevelopmental treatment 10 weeks, 4 sessions per week, 30 min per session
Fernandes, LC et al., 2018 (India) [67] Setting: K.L.E.S Hospital and MRC, Belgaum, Karnataka Funding: No	Spastic diplegic CP	I–III	30	15	6.9 ± 1.9 years old	8:7	HRS plus Conventional therapy 6 weeks, 3 sessions per week, 60 min per session (30 min conventional therapy plus 30 min HRS)	15	7.5 ± 2 years old	7:8	Conventional therapy 6 weeks, 3 sessions per week, 60 min per session
Hemachithra, C et al., 2020 (India) [68] Setting: Physical Medicine and Rehabilitation, Rajah Muthiah Medical College Hospital, Annamalai University Funding: No	Spastic diplegic CP	I–III	24	12	2–4 years old	6:6	HRS One session, 30 min per session	12	2–4 years old	6:6	Sham One session, 30 min per session
Herrero, P et al., 2012 (Spain) [69] Setting: Schools run by the Department of Education of the Government of Aragon, Spain. Funding: Aragon Government: PM059/2007	Spastic diplegic CP	I–IV	38	19	9.95 ± 0.6 years old	5:14	HRS 10 weeks, 1 session per week, 15 min per session	19	9.05 ± 0.7 years old	9:10	Sham 10 weeks, 1 session per week, 15 min per session
Jung, YG et al., 2022 (South Korea) [70] Setting: Samsung Changwon Hospital Funding: No	Spastic diplegic CP	I–IV	17	10	9.33 ± 2.1 years old	3:7	HRS plus Conventional therapy 8 weeks, 2 sessions per week, 30 min per session	7	9.08 ± 2.4 years old	3:4	Conventional therapy plus home bases aerobic exercise 8 weeks, 2 sessions per week
Kang, KY et al., 2010 (South Korea) [71] Setting: NR Funding: No	Spastic diplegic CP	III–IV	24	12	10.5 ± 2.9 years old	6:6	HRS plus Conventional therapy 12 weeks, 3 sessions per week, 45 min per session (30 min conventional therapy plus 15 min HRS)	12	9.08 ± 2.1 years old	5:7	Conventional therapy 12 weeks, 3 sessions per week, 30 min per session
Quint, C et al., 1998 (USA) [72] Setting: The Lord Mayor Treloar School. Alton, Illinois. Funding: No	Spastic diplegic CP	NR	30	15	9–16 years old	NR	HRS 4 weeks, 10 times during the school day, 10 min per session	15	9–16 years old	NR	Sham 4 weeks, 10 times during the school day, 10 min per session
Silva-Borges, MB et al., 2011 (Brazil) [73] Setting: The Clinic of Physiotherapy and Laboratory of Biomechanics of the Catholic University of Brasilia Funding: No	Spastic diplegic CP	I–V	40	20	5.65 ± 2.48 years old	12:8	HRS 6 weeks, 2 sessions per week, 40 min per session	20	5.77 ± 2.3 years old	11:9	Conventional therapy 6 weeks, 2 sessions per week, 40 min per session
Temcharoensuk, P et al., 2015 (Thailand) [74] Setting: Rehabilitation Centre, Mahidol Funding: No	Spastic diplegic CP	I–III	20	10	10.1 ± 1.7 years old	6:4	HRS 30 min per session	10	10.4 ± 1.5 years old	5:5	Sham 30 min per session

Abbreviations: CP, cerebral palsy; GMFCS, gross motor function classification system; N, sample size; N_e_, number of participants in experimental intervention; N_c_, number of participants in control intervention; F, female; M, male; NR, not reported; HRS, horse-riding simulator; Min, minutes.

**Table 3:** Cochrane Risk of Bias Tool scores for studies included in the review.

Study	Selection Bias		Performance Bias	Detection Bias	Attrition Bias	Reporting Bias	Other Bias
	Random Sequence Generation	Allocation Concealment	Blinding of Participants	Blinding of Assessors	Incomplete Outcome Data	Selective Reporting	Anything Else, Ideally Pre-Specified
Bagheri, H et al., 2017 [63]	−	−	+	+	−	?	−
Chinniah, H et al., 2020 [64]	−	−	+	+	−	?	−
Choi, HJ et al., 2014a [65]	−	+	+	+	−	?	−
Choi, HJ and Nam, KW 2014b [66]	−	+	+	+	−	?	−
Fernandes, LC et al., 2018 [67]	−	+	+	+	−	−	?
Hemachithra, C et al., 2020 [68]	−	−	+	−	−	?	−
Herrero, P et al., 2012 [69]	−	−	+	−	−	−	−
Jung, YG et al., 2022 [70]	?	?	+	+	−	−	?
Kang, KY et al., 2010 [71]	−	+	+	+	−	−	?
Quint, C et al., 1998 [72]	−	−	+	−	−	−	?
Silva−Borges, MB et al., 2011 [73]	−	+	+	−	−	−	?
Temcharoensuk, P et al., 2015 [74]	−	+	+	−	−	−	?

Abbreviations: “+,” high risk of bias; “−,” low risk of bias; “?,” uncertain risk of bias.

**Table 4:** Qualitative synthesis of the findings.

Study	Outcomes		
	Variable	Test	Qualitative Findings
Bagheri, H et al., 2017 [63]	GMF (Total)	GMFM-66	Statistically significant differences in HRS plus strength training and HRS plus conventional therapy groups (*p* = 0.021 and *p* = 0.001 respectively), but not in conventional therapy alone (*p* = 0.156)
	Functional balance	PBS	No statistically significant differences in all groups (*p* > 0.05)
	Adductors spasticity	MMAS	No significant differences were found between groups and within groups (*p* > 0.05)
	Knee flexors spasticity	MMAS	No significant differences were found in each group (*p* < 0.05)
	Ankle plantar flexors spasticity	MMAS	Statistically significant differences in HRS plus strength training group in right ankle plantar flexors strength (*p* = 0.05)
Chinniah, H et al., 2020 [64]	GMF (B Dimension)	GMFM-88	Both groups reported significant improvements (*p* < 0.001, respectively). The experimental groups show higher mean values than the control group. Statistically significant differences were found between groups (*p* = 0.028). Interaction analysis showed more improvement in the experimental group than the control group in each comparison (week assessment).
Choi, HJ et al., 2014a [65]	Posturographic spinal posture	ABW Mapper	Statistically significant differences in interaction between groups and periods in trunk imbalance, pelvic torsion and pelvic tilt (*p* < 0.05).
Choi, HJ and Nam, KW 2014b [66]	Posturographic static balance	Pedoscan sensor	No significant differences in interaction between the group and period (*p* > 0.05). Statistically significant differences between groups in ML sway (*p* < 0.05) No significant differences between groups and within groups in AP sway (*p* > 0.05).
Fernandes, LC et al., 2018 [67]	Functional balance	PBS	Statistically significant differences in both groups (*p* < 0.0001 respectively). No statistically significant differences were found between groups in the post-intervention assessment (*p* = 0.4516)
	GMF (Total)	GMFM-66	Statistically significant differences in both groups (*p* < 0.0001 respectively). No statistically significant differences were found between groups in the post-intervention assessment (*p* = 0.4516)
Hemachithra, C et al., 2020 [68]	Adductors spasticity	MAS	Statistically significant differences in the experimental group (*p* < 0.001). Statistically significant differences between groups (*p* < 0.001)
	Hip ROM	Goniometry	Statistically significant differences in the experimental group (*p* < 0.001). Statistically significant differences between groups (*p* < 0.001)
Herrero, P et al., 2012 [69]	GMF (Total and B dimension)	GMFM-66	Both groups improved, although the HRS group reported greater scores than the control group in sitting and total GMFM
Jung, YG et al., 2022 [70]	GMF (Total and A, B, C, D and E dimensions)	GMFM-88	For A, B and C dimensions, no statistically significant differences between groups and within groups (*p* > 0.05). For the D dimension, statistically significant differences were found in HRS (*p* = 0.03) but not between groups (*p* = 0.06). For the E dimension, statistically significant differences were found in HRS (*p* =0.03) but not between groups (*p* = 0.19). For GMFM total score, statistically significant differences in the HRS group (*p* < 0.01) and between groups favors HRS (*p* < 0.01)
Kang, KY et al., 2010 [71]	GMF (Total and A, B, C, D and E dimensions)	GMFM-88	Statistically significant differences in A and B dimensions in the control group (*p* = 0.04 and *p* = 0.019, respectively). Statistically significant differences in all items in the HRS group (*p* < 0.05). Significant differences between groups favor HRS in the C, D and E dimensions (*p* = 0.04, *p* = 0.047 and *p* = 0.049, respectively).
Quint, C et al., 1998 [72]	Pelvic ROM	Goniometry	Both groups improved, although the experimental group reported a greater pelvic ROM after the intervention.
Silva-Borges, MB et al., 2011 [73]	Postural control (AP and ML)	F-mat sensor platform and F-scan system.	Statistically significant differences between groups favor the experimental group (*p* < 0.0001) in AP and ML displacement.
	Satisfaction	AUQEI	Scores were higher on the “physiotherapy” item in the HRS group, finding statistically significant differences (*p* = 0.0026). No child was unhappy with the use of the simulator, while 25% of children belonging to the CT group were unhappy with the therapy.
	GMF (Sitting ability	GMFCS	Statistically significant differences in the HRS group (*p* = 0.0110). No between groups
Temcharoensuk, P et al., 2015 [74]	GMF	GMFM-66	No statistically significant differences in both groups (*p* > 0.05). No statistically significant differences between groups (*p* > 0.05)
	Seated trunk control	SATco	Statistically significant differences in all groups. HR group reported more items with significant differences. The “Reactive control” item was statistically significant among the three groups (*p* < 0.05). Statistically significant differences were found in “reactive control” in the HR group vs. the SHS group comparison (*p* = 0.004).

Abbreviations: HRS, Horse-Riding Simulator; GMF, Gross Motor Function; GMFM-66, gross motor function measure-66; PBS, pediatric balance scale; MMAS, modified, modified Ashworth scale; GMFM-88, gross motor function measure-88; ROM, range of motion; GMFCS, gross motor function classification system.

**Table 5:** Main Findings in meta-analyses.

		Findings Summary											Quality Evidence (Grade)					
		Effect Size						Heterogeneity		Publication Bias								
		K	N	N_s_	SMD	95% CI	*p*	Q (df)	I^2^ *(p)*	Egger *p*	Trim and Fill		Risk of Bias	Incons	Indirect	Imprec	Publ. Bias	Quality
											Adj SMD	% Var						
GMF (A dimension)	HRS + PT vs. PT	2	41	20.5	0.25	−0.28–0.79	0.353	0.01 (1)	0% (0.92)	NP	NP	NP	Mod.	No	No	Yes	Prob.	Very low
GMF (B dimension)	Overall	5	129	25.8	0.52	0.15–0.9	0.006	9.65 (4)	41% (0.04)	0.07	0.6	15%	Mod.	Mod.	No	Yes	Yes	Very low
	HRS + PT vs. PT	3	71	23.6	0.84	0.32–1.36	0.002	3.5 (2)	42% (0.17)	0.62	0.84	0%	Mod.	Mod.	No	Yes	No	Low
	HRS vs. sham	2	58	29	0.19	−0.34–0.72	0.49	0.004 (1)	0% (0.94)	NP	NP	NP	Mod.	No	No	Yes	Prob.	Very low
GMF (C dimension)	HRS + PT vs. PT	2	41	20.5	0.19	−0.42–0.81	0.54	0.09 (1)	0% (0.76)	NP	NP	NP	Mod.	No	No	Yes	Prob.	Very low
GMF (D dimension)	HRS + PT vs. PT	2	41	20.5	0.32	−0.31–0.94	0.32	0.338 (1)	2% (0.56)	NP	NP	NP	Mod.	No	No	Yes	Prob.	Very low
GMF (E dimension)	HRS + PT vs. PT	2	41	20.5	0.13	−0.48–0.75	0.67	0.04 (1)	0% (0.84)	NP	NP	NP	Mod.	No	No	Yes	Prob.	Very low
GMF (Total)	Overall	7	187	26.7	0.64	0.34–0.94	<0.001	6.72 (6)	10.7% (0.35)	0.13	0.64	0%	Mod.	Low	No	Yes	No	Mod.
	HRS + PT vs. PT	5	109	21.4	0.98	0.35–1.62	0.002	4.85 (4)	17.6% (0.31)	0.08	1.11	13%	Mod.	Low	No	Yes	Yes	Low
	HRS vs. PT	1	40	40	0.41	−0.82–1.64	0.52	0 (0)	0%	NP	NP	NP	Mod.	No	No	Yes	Prob.	Very low
	HRS vs. sham	1	38	38	0.15	−1.01–1.4	0.8	0 (0)	0%	NP	NP	NP	Mod.	No	No	Yes	Prob.	Very low
Funct. balance	HRS + PT vs. PT	3	68	22.7	0.6	0.1–1.08	0.018	0.43 (2)	0% (0.8)	0.81	0.6	0%	Mod.	No	No	Yes	No	Low
Abduction pelvic ROM	HRS vs. sham	2	54	27	0.79	0.21–1.37	0.008	2.24 (1)	37% (0.13)	NP	NP	NP	Mod.	Mod.	No	Yes	Prob.	Very low
Spasticity hip add	Overall	3	62	20.7	−0.4	−0.92–0.11	0.122	2.06 (2)	3.05 (0.36)	0.19	−0.81	100%	Mod.	No	No	Yes	Yes	Low
	HRS + PT vs. PT	2	38	19	−0.15	−0.8–0.5	0.642	0.53 (1)	0% (0.46)	NP	NP	NP	Mod.	No	No	Yes	Prob.	Very low
	HRS vs. sham	1	24	24	−0.82	−1.65–0.02	0.054	0 (0)	0%	NP	NP	NP	Mod.	No	No	Yes	Prob.	Very low
Spasticity ankle flex	HRS + PT vs. PT	2	38	19	−0.55	−1.22–0.12	0.11	0.03 (1)	0% (0.86)	NP	NP	NP	Mod.	No	No	Yes	Prob.	Very low
Spasticity knee ext	HRS + PT vs. PT	2	38	19	−0.12	−0.77–0.52	0.71	2.73 (1)	57% (0.09)	NP	NP	NP	Mod.	Large	No	Yes	Prob.	Very low

Abbreviations: K, number of comparisons; N, sample size; N_s_, participants per comparison; SMD, standardized mean difference; 95% CI, 95% confidence interval; *p*, *p*-value; Q, Q-test; df, degree of freedom; I^2^, degree of inconsistency; Adj, adjusted; % var; % of change; Incons, inconsistency; Indirect, indirectness; Imprec, imprecision; Publ, publication; GMF, gross motor function, HRS, horse-riding simulator; PT, physiotherapy; NP, not possible; Mod, moderate; Prob, probably; Funct, functional; ROM, range of motion; Add, adductors; Flex, flexors; Ext, extensors.

## Data Availability

Not applicable.

## Supplementary Materials

The following supporting information can be downloaded at: https://www.mdpi.com/article/10.3390/bioengineering9120790/s1, Figure S1. Funnel plot for Gross Motor Function, Dimension B (overall meta-analysis); Figure S2. Funnel plot for Gross Motor Function, Total score (HRS plus PT vs. PT meta-analysis); Figure S3. Funnel plot for Spasticity Hip Adductors (overall meta-analysis.

## Abbreviations

HRS	Mechanical Horse-Riding Simulator
CP	Cerebral Palsy
PEDro	Physiotherapy Evidence Database
ROM	Range of Motion
SMD	Standardized Mean Difference
95% CI	95% Confidence Interval
PRISMA	Preferred Reporting Items for Systematic Reviews and Meta-Analyses
AMSTAR	A Measurement Tool to Assess Systematic Reviews
WOS	Web of Science
GRADE	Grading of Recommendations Assessment, Development and Evaluation
MD	Mean Difference
MCID	Minimally Clinically Important Difference
I^2^	Degree of Inconsistency
PT	Physiotherapy
GMFM	Gross Motor Function Measure
GMFCS	Gross Motor Function Classification System
PBS	Pediatric Balance Scale
MMAS	Modified Modified Ashworth Scale
MAS	Modified Ashworth Scale
AUQEI	Autoquestionnaire Qualité de Vie Enfant Image
