# Supplementary material for: Effectiveness of Mechanical Horse-Riding Simulator-Based Interventions in Patients with Cerebral Palsy—A Systematic Review and Meta-Analysis
Source: Bioengineering (Basel). 2022 Dec 11;9(12):790. doi: 10.3390/bioengineering9120790 (PMC9774556; doi:10.3390/bioengineering9120790)
Supplement: Supplementary file 1 [file bioengineering-09-00790-s001.zip › bioengineering-2052258-supplementary.pdf]

SUPPLEMENTARY FILES

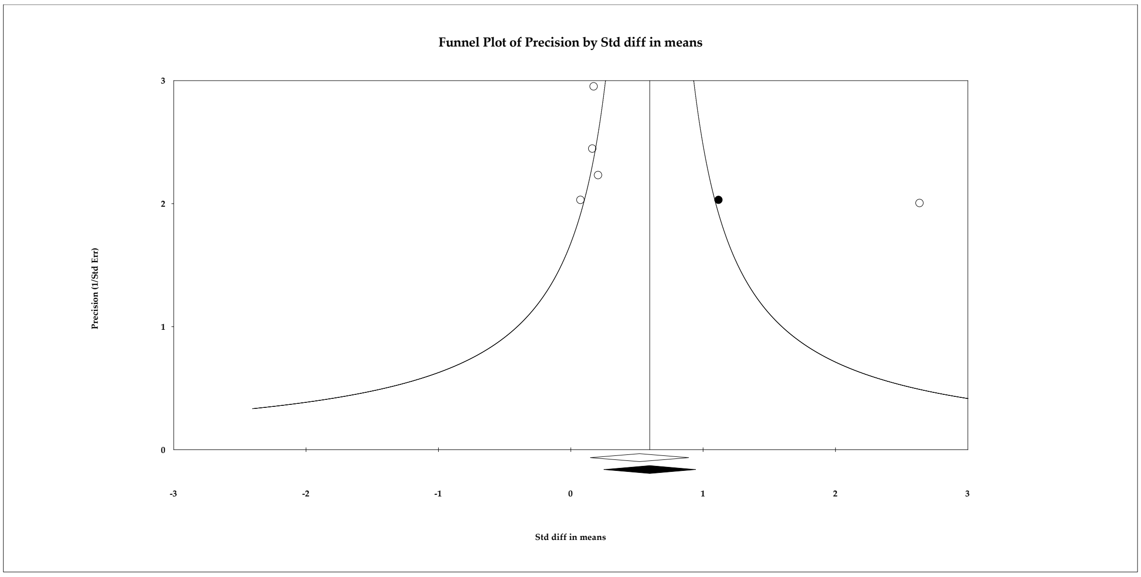

Figure S1. Funnel plot for Gross Motor Function, Dimension B (overall meta-analysis).

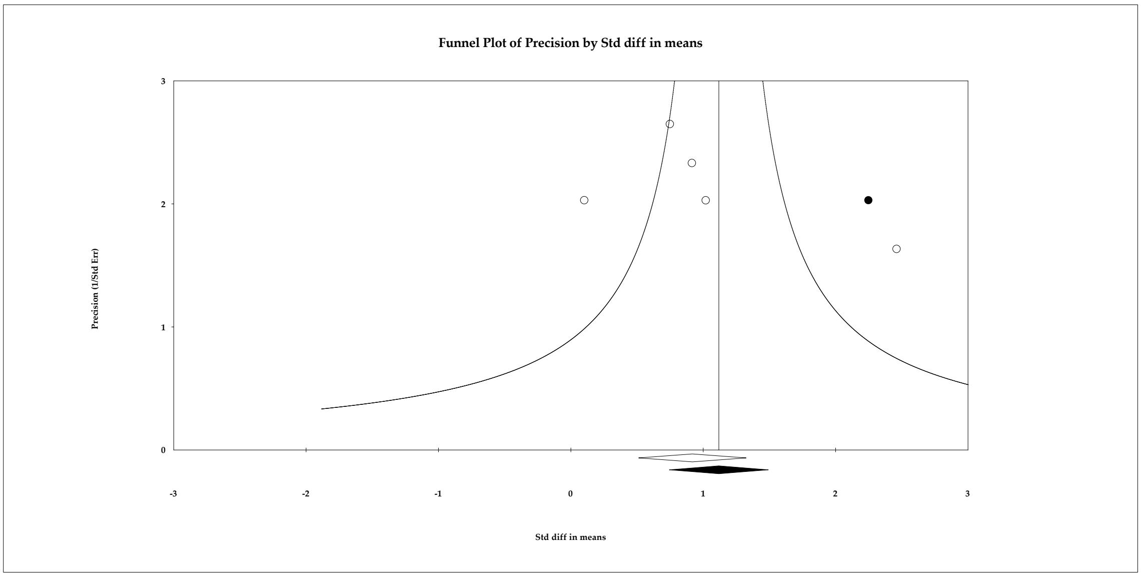

Figure S2. Funnel plot for Gross Motor Function, Total score (HRS + PT vs PT meta-analysis).

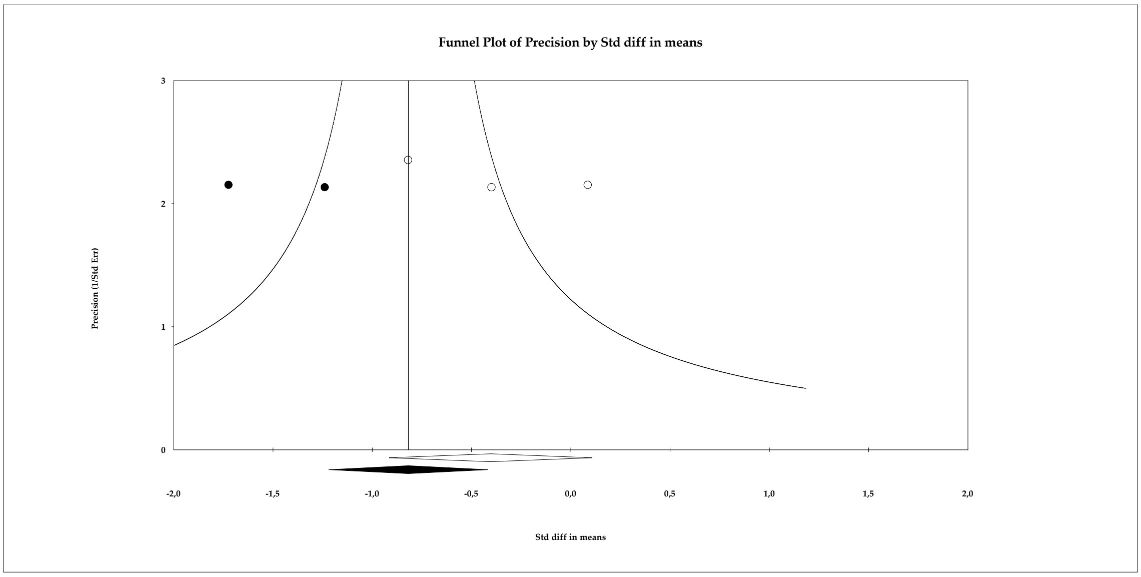

Figure S3. Funnel plot for Spasticity hip adductors (overall meta-analysis).
